# Domestic Mite Antigens in Floor and Airborne Dust at Workplaces in Comparison to Living Areas: A New Immunoassay to Assess Personal Airborne Allergen Exposure

**DOI:** 10.1371/journal.pone.0052981

**Published:** 2012-12-21

**Authors:** Ingrid Sander, Eva Zahradnik, Gerhard Kraus, Stefan Mayer, Heinz-Dieter Neumann, Christina Fleischer, Thomas Brüning, Monika Raulf-Heimsoth

**Affiliations:** 1 Institute for Prevention and Occupational Medicine of the German Social Accident Insurance, Institute of the Ruhr University Bochum (IPA), Bochum, Germany; 2 German Social Accident Insurance Institution for the energy, textile, electrical and media products sectors (BGETEM), Augsburg, Germany; 3 German Social Accident Insurance Institution for the trade and distribution industry (BGHW), Mannheim, Germany; 4 German Social Accident Insurance Institution for the public sector in North Rhine-Westphalia, Düsseldorf, Germany; Leiden University Medical Center, The Netherlands

## Abstract

**Objectives:**

Allergens produced by domestic mites (DM) are among the most common allergic sensitizers and risk factors for asthma. To compare exposure levels between workplaces and living areas a new assay able to measure airborne DM antigen concentrations was developed.

**Methods:**

At workplaces and in living areas, 213 floor dust samples and 92 personal inhalable dust samples were collected. For sensitive quantification of DM antigens, a new enzyme immunoassay (EIA) based on polyclonal antibodies to *Dermatophagoides farinae* extract was developed. Reactivity of five house dust mite and four storage mite species was tested. All dust samples were tested with the new EIA and with the Der f 1 and Der p 1-EIAs (Indoor Biotechnologies, UK) which detect major allergens from *D. farinae* and *D. pteronyssinus* by monoclonal antibodies. Samples below the detection limit in the DM-EIA were retested in an assay variant with a fluorogenic substrate (DM-FEIA).

**Results:**

The newly developed DM-EIA detects antigens from all nine tested domestic mite species. It has a lower detection limit of 200 pg/ml of *D.farinae* protein, compared to 50 pg/ml for the DM-FEIA. DM antigens were detected by DM-EIA/FEIA in all floor dust and 80 (87%) of airborne samples. Der f 1 was found in 133 (62%) floor dust and in only 6 airborne samples, Der p 1 was found in 70 (33%) of floor samples and in one airborne sample. Der f 1 and DM concentrations were highly correlated. DM-antigens were significantly higher in inhalable airborne samples from textile recycling, bed feather filling, feed production, grain storage and cattle stables in comparison to living areas.

**Conclusions:**

A new sensitive EIA directed at DM antigens was developed. DM antigen quantities were well correlated to Der f 1 values and were measurable in the majority (87%) of airborne dust samples. Some workplaces had significantly higher DM antigen concentrations than living areas.

## Introduction

Multicenter studies across Europe have found that house dust mites, among the indoor allergens, yield the highest sensitization frequency [1] and that exposure to mite allergens is an important risk factor for allergic respiratory diseases and asthma [2]–[5]. Therefore, many studies on mite allergen concentrations have been performed. In most cases, reservoir dust from floors or mattresses has been analysed by monoclonal antibody-based immunoassays to group I or II allergens from the house dust mites *Dermatophagoides pteronyssinus* and *D.farinae* [6]–[8]. However, according to Paufler et al. [9] “assessing allergens in ambient air would better represent human exposure because inhalation is the main route of uptake, and a close correlation between levels of floor and air antigens has not yet been proved”. Especially for occupational allergen exposure assessment, the method of choice is the sampling of inhalable dust in the breathing zone of the suspected exposed worker and then the determination of allergen levels per cubic meter. Due to extremely low allergen-carrying particle concentrations, airborne exposure assessment of mite allergens is difficult to perform. In several studies, the level of airborne house dust mite allergens in undisturbed conditions could not be determined because the limits of detection of the assays used were inadequate [10]. Therefore, the first step for measuring airborne mite allergen exposure was to develop an assay with sufficient sensitivity.

To assess for allergens besides mite group I or group II allergens, polyclonal antibodies directed at an allergen extract from *D. farinae* were used for a two-sited EIA amplified by a poly-enzyme-conjugate and, in the most sensitive assay variant, by a fluorogenic substrate. The specificity of the new assay was controlled by testing seed, mould, insect and other mite extracts and by comparison with the results of the Der f 1 and Der p 1 assay. In this pilot study, different workplaces with suspected mite exposure were compared with living areas; in total, 213 floor dust samples and 92 personal inhalable dust samples were collected and analysed.

## Materials and Methods

### Mites, seeds and moulds

The mite allergens used for our study were obtained from ALK-Albelló, Madrid, Spain, with the exception of *D. farinae* and *D. pteronyssinus*, which were obtained from Allergopharma, Reinbek, Germany. *Blattella germanica* and *Periplaneta americana* in the form of lyophilized dry material were obtained from Greer, Lenoir, USA, *Sitophilus granarius*, *Ephestia kuehniella*, and *Tribolium confusum* imagines were a gift from the Julius Kühne Institut, Berlin, Germany and *Tenebrio molitor* larvae were bought from a pet shop. Moulds in the form of lyophilized dry material were obtained from Allergon, Ängelholm, Sweden. Plant seeds were bought from a health food shop. Protein concentrations of all extracts were measured by a Bradford assay (Bio-Rad, München, Germany) with bovine serum albumin as a standard.

### Production, purification and biotinylation of antibodies

The animal experiments were approved by the local commissioner for animal welfare and notified by the “Landesamt für Natur, Umwelt und Verbraucherschutz, Nordrhein-Westfalen”. A female New Zealand white rabbit was immunized subcutaneously with 0.5 mg of *D. farinae* extract emulsified in TiterMax Gold (TiterMax® USA, Norcross, GA, USA). Two booster injections with the same allergen concentration in TiterMax Gold were carried out at 5-week intervals. The serum was collected 5 weeks after the last injection and stored at −80°C. For antibody purification, the IgG fraction was isolated by affinity chromatography with HiTrap Protein A column and with antigen coupled N-hydroxysuccinimide-ester-activated Sepharose column following the manufacturer's instructions (GE Healthcare, Uppsala, Sweden). Antigen-affinity purified antibodies were biotinylated by mixing with a 30-fold molar excess of biotinoyl-ε-aminocaproic acid-N-hydroxysuccinimide ester (Roche, Mannheim, Germany) dissolved in dimethylsulfoxide for 4 h at room temperature.

### Domestic mite sandwich EIA

MaxiSorp microtiter plates (Nunc, Roskilde, Denmark) were coated overnight at 4°C with Protein A-affinity purified antibodies at 0.4 µg/ml in 0.1 M carbonate/bicarbonate buffer, pH 9.6. After 2 h blocking with 1.5% (w/v) casein in phosphate-buffered saline with 0.05% Tween 20 (PBST), the microwells were incubated with standards, assay controls and samples diluted in PBST for 1 h at 22°C. A standard curve was obtained using serial 1/2 dilutions from 10–0.04 ng/ml of the *D. farinae* extract (Allergopharma). House dust extract was used as a positive control, and wheat flour extract was used as a negative control. Each sample was tested at three serial 1/2 dilutions. The captured mite proteins were detected with antigen-affinity purified and biotinylated antibodies (0.1 µg/ml, 1 h, 22°C), followed by streptavidin-peroxidase conjugate (Poly-HRP80-SA, Fitzgerald, Concord, MA, USA) (1/10,000 in PBST, 1 h, 22°C) and finally ABTS substrate solution [2,2′-azino-bis(3-ethylbenzothiazoline-6-sulfonic-acid)] diammonium salt, Sigma-Aldrich, Steinheim, Germany) in 50 mM phosphate-citrate buffer pH 4.2 with 0.015% H_2_O_2_. The reaction was stopped with 0.32% sodium fluoride, and the absorbance was read at 414 nm. Samples with mite concentrations below the lower limit of detection (LOD) were also analysed using an amplified EIA with QuantaBlu fluorogenic substrate (ThermoScientific, Rockford, IL, USA) instead of ABTS. Sample concentrations were calculated by interpolation of optical density (OD) values or relative fluorescence units on a 4-parameter fitted standard curve using Softmax Pro 5.4.1 (Molecular Devices, Sunnyvale, CA, USA). The LOD was the concentration corresponding to OD  = 0.1 or 600 relative fluorescence units (∼5 times the standard deviation of the background value) above the estimated minimum value (‘parameter A’) of the 4-parameter curve fit function.

### Der f 1 and Der p 1 EIAs

Der f 1 and Der p 1 allergens were quantified using monoclonal antibodies and detection kits (EL-DF1, EL-DP1) from Indoor Biotechnologies Ltd. (Charlottesville, VA, USA) according to the manufacturer's instructions. The universal standard was used in a concentration range of 25–0.1 ng/ml Der f 1, and 125–0.5 ng/ml Der p 1, respectively. The LOD was the concentration corresponding to OD  = 0.02 (∼5 times the standard deviation of the background value) above the estimated minimum value (‘parameter A’) of the 4-parameter curve fit function. The average LOD of Der f 1 assay was 0.15 ng/ml, the average LOD of Der p 1 assay was 1.05 ng/ml.

### Immunoblotting

Mite extracts (5 µg protein per lane) were separated by sodium dodecyl sulfate polyacrylamide gel electrophoresis (SDS-PAGE) and blotted as described previously [11]. The blocked membranes were incubated overnight with rabbit anti-*D. farinae* serum (diluted 1/10,000) or with human IgE anti-mite serum pool (diluted 1/5). For the serum pool, ten sera (*D. farinae* IgE > *D. pteronyssinus* IgE, all CAP class 4 17.5–50 kU_A_/L by ImmunoCAP, Phadia, Freiburg, Germany) were mixed, all derived from house dust mite-sensitized patients who gave informed consent. Alkaline phosphatase-conjugated anti-rabbit IgG (1/30,000, Sigma-Aldrich) or anti-human IgE (1/1000, Sigma-Aldrich) were used as secondary antibodies, and bound antibodies were detected as described previously [12].

### Sampling and extraction of floor dust and inhalable dust

Sampling took place in Germany from mid-2007 until the end of 2011. Floor dust samples were taken in sleeping rooms (n = 56), living and working rooms (n = 20), kitchens (n = 4) and corridors (n = 4) of 60 private homes and in 21 workplaces of 11 different industries (n = 129). Permission for sampling at workplaces was obtained by the employers and involved employees to obtain information on house dust mite allergen concentrations, sampling in own private homes was performed by volunteering colleagues of the authors.

ALK dust collection cassettes (ALK-Abelló, Hørsholm, Denmark) with pre-weighed glass fiber filters (Macherey & Nagel, Düren, Germany) were attached on vacuum cleaners, and an area of 1–2 m^2^ on floors was vacuumed for 4 minutes. Within the next three days, samples were frozen (−20°C) for 24 h to kill mites and subsequently stored at room temperature until extraction.

Personal airborne dust samples were taken at 33 workplaces of 15 different industries (n = 76) and in 16 living areas during housework including vacuuming and bed-making (n = 16) for 1–4 h with GSP-sampling heads for inhalable dust and air samplers at a flow rate of 3.5 l/min in the individuals' breathing zones. All sampling was performed with pre-weighed and coded 3.7 cm ∅ polytetrafluoroethylene (PTFE) filters with 1 µm pore size (FALP03700, Millipore). After sampling and conditioning for 24 h, the dust load of the filters was weighed. Three reference filters for vacuuming or airborne dust were pre- and reweighed at each weighing. They were used to calculate the mean plus three times the standard deviation to estimate the detection limits for weighing. For floor dust, 118 blank values gave a detection limit of 0.41 mg per filter, and all floor filters contained weighable dust amounts. The 90 blank values of PTFE reference filters resulted in a detection limit of 0.21 mg per filter (0.5 mg/m^3^ for 2 h sampling). Filters with floor dust were extracted in 10–50 ml PBST, and PTFE filters were extracted in 5 ml PBST by mixing. All extracts were centrifuged for 15 min at 3000x *g*, and the supernatants were stored frozen in aliquots at −80°C until testing in mite assays.

### Statistics

The strength of the relationship between EIA measures was analysed using Pearson correlations. All weights or EIA values below the detection limit were replaced by 2/3 of this limit. The Mann-Whitney test was used for the comparison of workplaces with living areas. A pvalue of less than 0.05 was considered statistically significant. All statistical analyses were performed using GraphPad Prism (version 5.03 for Windows; GraphPad Software, San Diego USA, www.graphpad.com).

## Results

**Figure 1:**
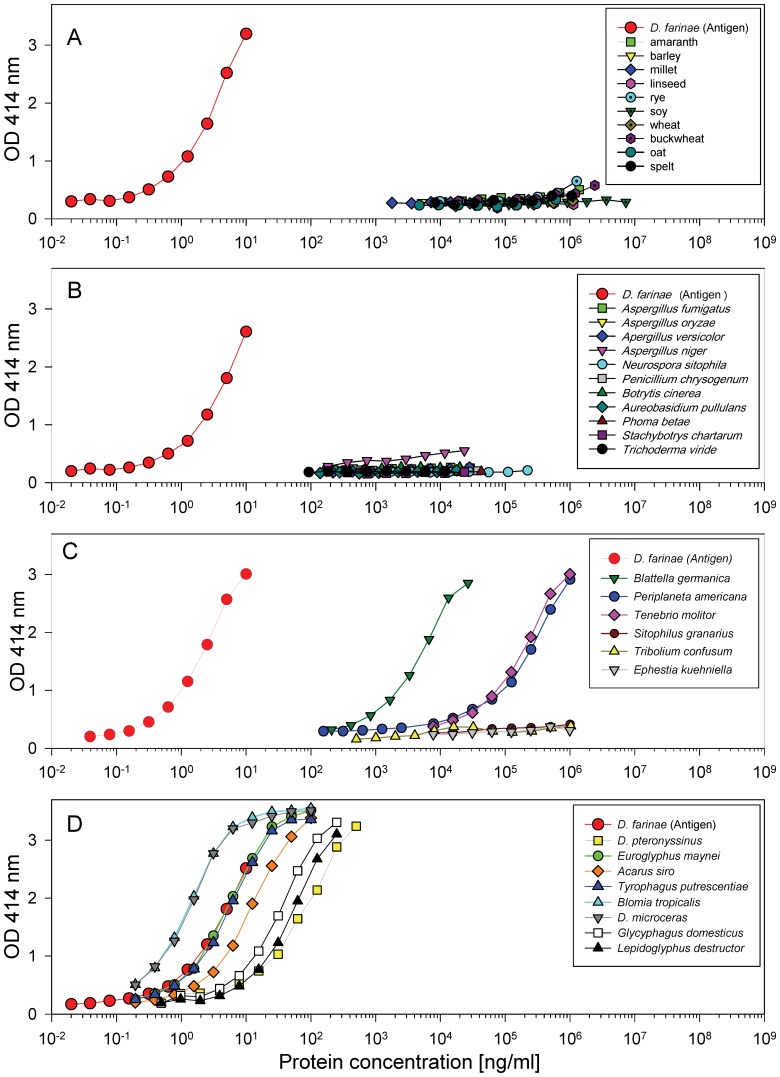
Specificity of the domestic mite EIA. Extracts from various plant seeds (A), moulds (B), insects (C) and several astigmata (D) were measured with the *D. farinae* derived polyclonal antibody based EIA. Concentrations are based on protein content.

**Figure 2:**
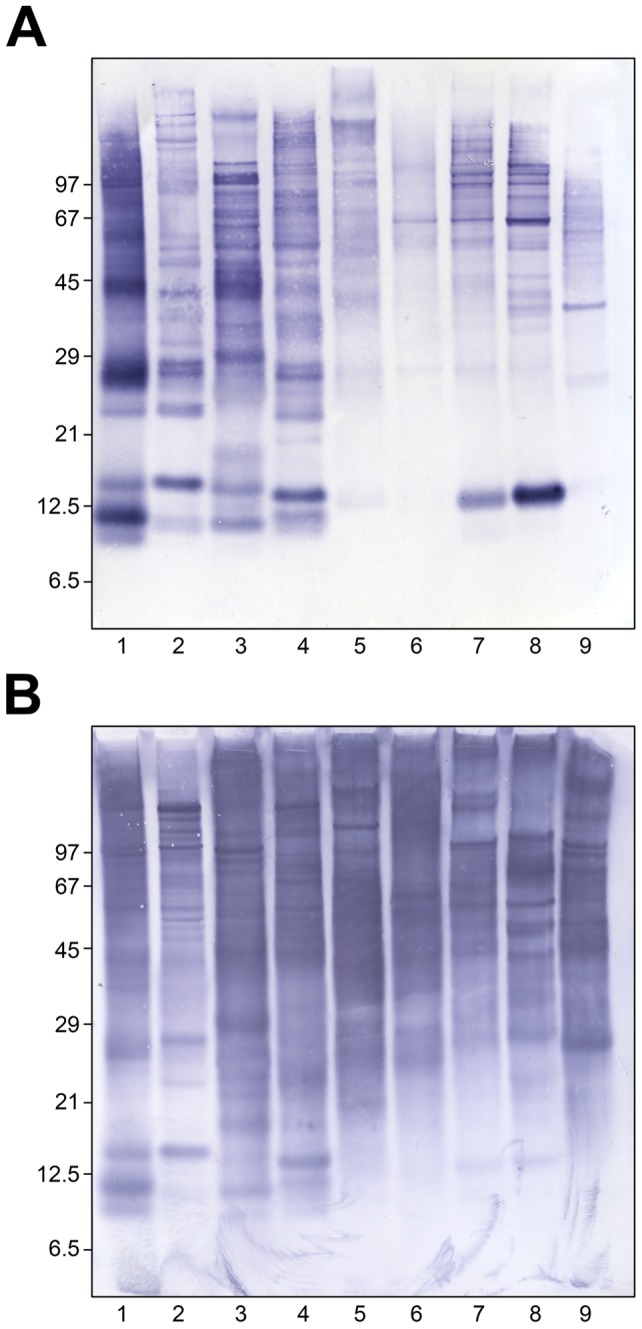
Immunoblots of domestic mites with human and rabbit antibodies. Extracts of the following domestic mite species were separated by SDS-PAGE: (1) D. *farinae*, 2 *D. pteronyssinus*, 3 *D. microceras*, 4 *Euroglyphus maynei*, 5 *Blomia tropicalis*, 6 *Acarus siro*, 7 *Lepidoglyphus destructor*, 8 *Glycyphagus domesticus*, 9 *Tyrophagus putrescentiae*. Immunoblots with pooled sera of mite sensitized patients and detection of specific IgE (A) and with the *D. farinae* immunized rabbit serum (B) were performed. The patients' pool serum had the following specific IgE concentrations (1: 39.9 kU_A_/L, 2: 27.7 kU_A_/L, 3: 30.8 kU_A_/L, 4: 5.34 kU_A_/L, 5: 2.3 kU_A_/L, 6: 2.95 kU_A_/L, 7: 3.49 kU_A_/L, 8: 3.02 kU_A_/L, 9: 3.39 kU_A_/L, order of mite species same as above).

With the purified polyclonal antibodies directed to antigens of the mite species *Dermatophagoides farinae*, an EIA was developed with a lower detection limit of 200 pg/ml. To assess the specificity of the assay, extracts from several materials were also tested. No reactivity was observed with seeds and grains or mould extracts (Fig. 1 a, b). Recovery rates of 1–5 ng/ml *D. farinae* standard protein used as spike in 10 µg/ml mould or 100 µg/ml seed extract was between 86–116% without any extract dependent difference. Whereas extracts from *Sitophilus granarius, Tribolium confusum* and *Ephestia kuehniella* showed no reactivity, the *Blattella germanica, Periplaneta americana* and *Tenebrio molitor* extracts reacted, although for factors of 3200, 70000 and 60000 lower than *D. farinae* protein, respectively (Fig. 1c). In addition, extracts from eight different domestic mite species were tested (Fig. 1d). Highest reactivity was obtained with proteins from *Dermatophagoides microceras* and *Blomia tropicalis*, and the lowest reactivity was obtained with *D. pteronyssinus* (a factor of 24 lower than *D. farinae* protein). Reactivity of the polyclonal rabbit antibodies to different mite extracts was analysed in comparison to IgE binding of a patients' pool serum by immunoblots (Fig. 2). The patients' pool and the rabbit antibodies reacted to proteins from all mite species, although more protein bands were detected by rabbit antibodies than by human IgE. In contrast, IgE-binding to proteins of 15 kDa from *L. destructor* and *G. domesticus* showed stronger bands than binding by the rabbit antibodies.

**Figure 3:**
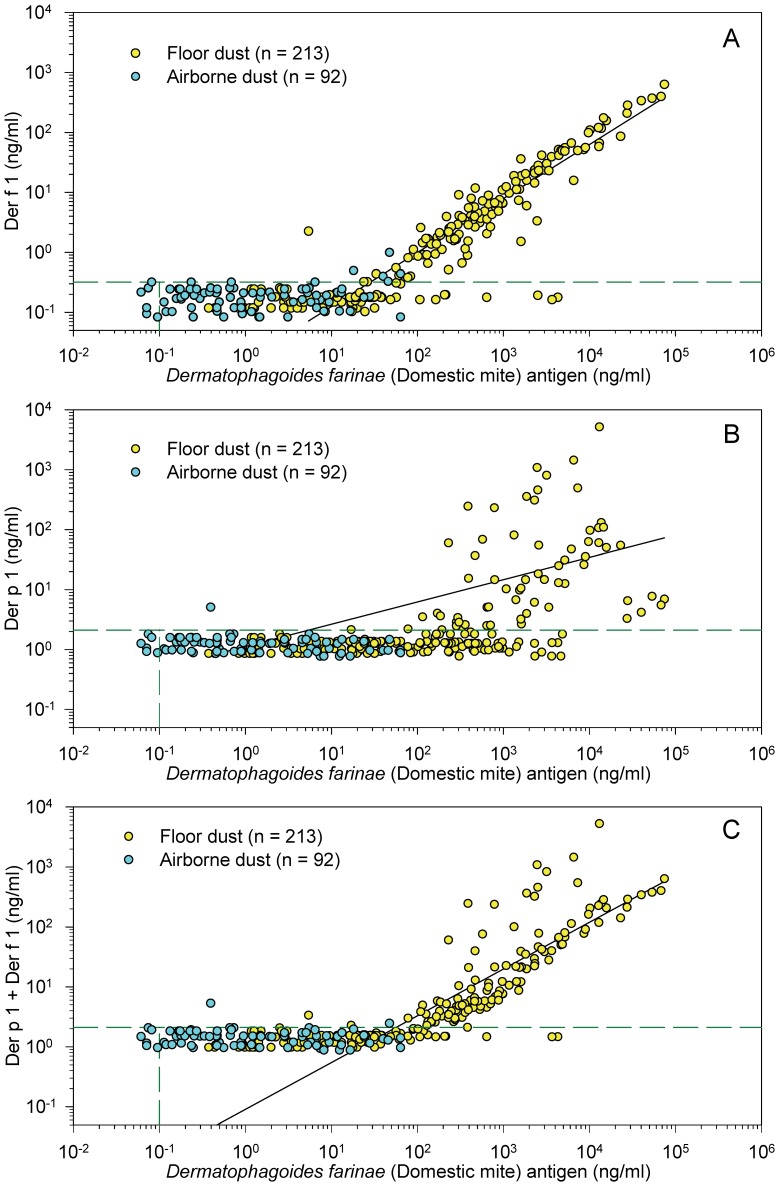
Comparison between the domestic mite and the Der f 1 (A), Der p 1 (B) or the sum of Der f 1 and Der p 1 (C) results in airborne dust or floor dust samples (dashed lines  =  detection limits, solid lines  =  regression lines of double positives).

As the newly developed *D. farinae* assay detects extracts of all other tested Astigmata, it was named “Domestic Mite EIA (DM-EIA)”. All floor dust and airborne samples were analysed with the DM-EIA and with the Der f 1 and Der p 1 EIAs. Whereas Der f 1 was found in 133 (62%) floor dust and only 6 airborne samples, and Der p 1 was measured in 70 (33%) floor dust and one airborne sample, all floor dust samples and 56 (61%) of the airborne samples were measurable with the DM-EIA. All samples with detectable Der f 1or Der p 1 allergen also contained domestic mite antigens. Sensitivity of the DM-EIA was further improved by using a fluorogenic substrate, resulting in a lower detection limit of 50 pg/ml. A comparison of 100 measurements above the detection limit of DM-EIA and DM-FEIA showed nearly identical values with both assay variants (mean ratio EIA versus FEIA value: 1.1±0.3). From 36 airborne samples negative in the DM-EIA, 24 were above the detection limit of the DM-FEIA. Thus, 80 (87%) of airborne samples contained measurable amounts of domestic mite antigens. Comparing the domestic mite antigen values with the Der f 1 values, a very high correlation for all samples (Pearson r = 0.97, Pearson correlation of log-transformed values r = 0.84) and of double positives (Pearson r = 0.97, Pearson correlation of log-transformed values r = 0.94) was observed (Fig. 3a). The domestic mite antigen content in double positives was at mean 144±121 times higher than the Der f 1 content. The standards of the Der f 1 EIA and DM-FEIA were also compared in a “cross-assay” design. The *D. farinae* standard of the DM-FEIA contained at mean 0.7% Der f 1 while the universal standard of the Der f 1 EIA which contains among other purified single allergens 2.5 µg/ml Der f 1 yielded a value of 2.4 µg/ml antigen in the DM-FEIA.

Comparing the domestic mite antigen (or Der f 1) values with the Der p 1 values, only a low to moderate correlation was observed (Fig. 3 b, Table 1). The correlation was increased when the sums of Der f 1 and Der p1 were compared to DM-values (Fig. 3 c, Table 1).

**Table 1:** Pearson correlation coefficients of Domestic mite, Der f 1 and Der p 1 EIA results from 213 floor dust samples (Pearson correlation coefficients of log-transformed values are printed in bold, Pearson correlation coefficients of untransformed values are printed in italics).

	DM	Der f 1	Der p 1	Der f 1+ Der p 1
	(ng/ml)	(ng/ml)	(ng/ml)	(ng/ml)
DM (ng/ml)		**0.90**	**0.58**	**0.83**
		*0.97*	*0.09*	*0.26*
Der f 1 (ng/ml)			**0.56**	**0.87**
			*0.05*	*0.22*
Der p 1 (ng/ml)				**0.86**
				*0.98*

In the floor dust samples from living areas, Der f 1 could be measured in 73 of 84 (87%) samples, and Der p 1 in 32 (38%) samples. Differences of Der f 1, Der p 1 and domestic mite antigen concentrations per gram floor dust were not significant between sleeping rooms and living/working rooms, although maximum concentrations were found in sleeping rooms. Comparing floor dust samples from living areas with workplaces, only textile recycling and bed feather cleaning resulted in higher median domestic mite antigen and Der f 1 concentrations (Table 2). All other workplaces had lower domestic mite and Der f 1 concentrations in dust than living areas. These differences were highly significant in most cases.

**Table 2:** Domestic mite antigens, Der f 1 and Der p 1 allergens in floor dust samples.

Business line	Sample number	Domestic mite antigens			Der f 1 allergen				Der p 1 allergen		
		Median	Range	p value*	>LOD	Median	Range	p value*	>LOD	Median	Range
	[n]	[ng/g]	[ng/g]		[n]	[ng/g]	[ng/g]		[n]	[ng/g]	[ng/g]
**Living areas**	84	24790	1101– 1217000		73	264	<LOD-10240		32	<LOD	<LOD-37240
Textile recycling	18	114200	14820– 37400	**0.0010**	18	1015	170–2012	**0.0013**	18	705	110–1260
Feather bed cleaning	4	69890	49450– 81250	0.1356	4	491	410–637	0.1763	1	<LOD	<LOD-160
Feather bed filling	28	2143	598– 19690	**<0.0001**	11	<LOD	<LOD-57	**<0.0001**	8	<LOD	<LOD-410
Grain storage	8	13990	2710– 21680	**0.0334**	0	<LOD		**<0.0001**	0	<LOD	
Tailoring	4	8145	3820– 29580	0.0639	4	64	39–346	0.1304	2	45	<LOD-50
Carpet/Upholstery cleaning	22	2682	435– 11730	**<0.0001**	20	22	<LOD-143	**<0.0001**	9	<LOD	<LOD-400
Furrier's workshop	8	805	350– 1921	**<0.0001**	0	<LOD		**<0.0001**	0	<LOD	
Toy distribution	4	499	186– 763	**0.0008**	2	3	<LOD-4	**0.0008**	0	<LOD	
Offices	3	677	535– 2641	**0.0047**	0	<LOD		**0.0161**	0	<LOD	
Schools	25	630	162– 7670	**<0.0001**	1	<LOD	<LOD-1339	**<0.0001**	0	<LOD	
Horticulture	5	16	11–35	**0.0002**	0	<LOD		**0.0002**	0	<LOD	

* P values were calculated by Mann-Whitney test in comparison to living areas. For Der p 1 the majority of measurements were below the detection limit, therefore no statistical comparison was performed.

Analyzing airborne samples, the comparison between living areas and workplaces showed a somewhat different picture (Table 3). At most workplaces, inhalable domestic mite concentrations were higher than those in living areas. These differences were significant for textile recycling, bed feather filling, feed production, grain storage and cattle stables. Inhalable airborne Der f 1 was measurable during textile recycling (five samples >LOD) and feather bed filling (one sample >LOD) alone (data not shown). Inhalable dust concentrations were significantly higher at nearly all workplaces than in living areas during house work.

**Table 3:** Personal airborne dust samples and Domestic mite antigens.

Business line	Sample number	Dust				Domestic mite antigen			
		>LOD	Median	Range	p value*	>LOD	Median	Range	p value*
	[n]	[n]	[mg/m^3^]	[mg/m^3^]		[n]	[ng/m^3^]	[ng/m^3^]	
**Living areas**	16	3	<LOD	<LOD-4.6		12	2.1	<LOD-38.6	
Textile recycling	12	12	2.1	0.35–6.6	**0.0001**	12	159.8	33.9–376.7	**<0.0001**
Feather bed filling	8	8	3.6	0.9–6.7	**0.0004**	8	37.3	1.1–142.1	**0.0092**
Feed production	4	4	3.4	3.1–3.9	**0.0079**	4	36.3	21.4–55.9	**0.0093**
Grain storage	5	4	0.4	<LOD-1.7	**0.0337**	5	27.2	3.5–95.4	**0.0349**
Cattle stables	17	13	1.0	<LOD-5.8	**0.0011**	16	8.8	<LOD-378.5	**0.0455**
Tea Packaging	1	0	<LOD			1	9.4		
Waste sorting	4	2	0.6	<LOD-2.6	**0.0298**	2	2.6	<LOD-33.5	0.4777
Tailoring	1	1	1.6			1	5.5		
Carpet/Upholstery cleaning	5	5	0.8	0.5–1.0	**0.0063**	5	4.4	1.7–52.3	0.2306
Furrier's workshop	3	3	0.4	0.3–0.4	0.0964	3	3	0.7–5.3	0.9553
Toy distribution	2	0	<LOD			2	1.5	1.2–1.8	
Bakery	2	2	26.4	18.8–34		2	1.3	1.2–1.5	
Slaughterhouse	6	6	0.8	0.5–1.1	**0.0031**	6	1.2	0.8–2.8	0.6053
Laboratory animal facility	6	3	0.4	<LOD-1.7	**0.0132**	1	<LOD	<LOD-2.8	0.5525

* P values were calculated by Mann-Whitney test in comparison to living areas. Mann-Whitney test requires at least three values in each group.

## Discussion

House dust mites provide the predominant inhalant allergens in many parts of the world, but geographic patterns for the common species *Dermatophagoides pteronyssinus* and *D. farinae* differ. Europe has mixed populations, with a trend of higher *D. pteronyssinus* allergen quantities in Western Europe near the coast and dominating *D. farinae* allergens in continental regions with cold winters. [8]. *D. farinae* populations increase with relative humidity of 50%, *D. pteronyssinus* increases at humidity over 60% [13]; thus, the most prevalent house dust mite in Germany varies from year to year. Low winter temperatures seem to reduce the major allergen Der p 1 from *D. pteronyssinus* rather than levels of Der f 1 from *D. farinae* [8]. In our study, 133 of the 213 floor dust samples contained Der f 1, whereas Der p 1 was detected in only 70 of these samples. One reason might have been the relative cold winters in Germany in 2008 to 2011. Thus, in our study, the house dust mite *D. farinae* was clearly the dominant species and therefore is the focus of the analysis presented here.

Using an allergen extract from *D. farinae* for immunization, a new sandwich EIA was developed with increased detection rate but lower specificity in comparison to the monoclonal antibody based Der f 1 assay from Indoor Biotechnologies. The EIA detects many antigens from *D. farinae* and from other Astigmata in the following order: *D. microceras, Blomia tropicalis, Euroglyphus maynei, Tyrophagus putrescentiae, Acarus siro, Glycyphagus domesticus, Lepidoglyphus destructor and D. pteronyssinus*. A second immunization with a *D. farinae* preparation from Allergon had been performed (data not shown). This *D. farinae* preparation contained 88% of the antigen and 38% of the Der f 1 content in comparison to the first immunogen. With the new antibodies again all other mite extracts reacted in nearly the same order. Thus, the reactivity pattern does not seem to be arbitrary but may be caused by the amount and similarities of immunodominant mite proteins which do not necessarily reflect the evolutionary relatedness of the species in all cases. Taking this broad detection pattern into account, the newly developed assay was named “domestic mite EIA”, as, according to Spieksma [14], “domestic mites' are free-living mites found regularly in the home environment and belonging to two ecologically defined groups: house-dust mite and storage mites.”

Whereas high concentrations of seeds and mould proteins did not influence the domestic mite EIA, very high concentrations of cockroach (*Blattella germanica* and *Periplaneta americana*) and mealworm (*Tenebrio molitor*) protein reacted, although for orders of magnitude less than domestic mites.

However, human IgE antibodies also have a broad detection pattern. Typically, most mite sensitized persons have IgE antibodies to both common house dust mites [15]–[17] and, to a lesser extent, to several storage mites [18], [19], and also in some cases to cockroaches [20] partially due to cross-reactivity of antibodies. The pool serum from ten house dust mite sensitized patients contained IgE antibodies to all tested domestic mite species and detected protein bands of all mite extracts in the IgE immunoblot. As the rabbit serum IgG immunoblot, the human IgE immunoblot showed many more mite allergens than group I (∼30 kDa in SDS-PAGE under reducing conditions) or group II (∼14 kDa) allergens. As the newly developed DM assay detects antigens of many domestic mite species comparable to the human IgE antibodies of domestic mite sensitized patients, its less specific detection profile in comparison to monoclonal antibody-based assays might not be a disadvantage for the assessment of health relevant exposure. Although the rabbit antibodies detect more proteins than the human IgE antibodies, and clearly more proteins than monoclonal antibodies to major allergens, for exposure assessment this problem can be handled as long as the measured antigen amount is proportional to the allergen amount.

Therefore, the concentrations of the major allergens Der f 1 and Der p1 were compared to the DM-antigen values. Despite the differences in specificities between the DM-EIA and the Der f 1 EIA, the measurement values were highly correlated (Pearson r = 0.97, Pearson of log-transformed floor dust values r = 0.90). Whereas the correlation of DM-antigen concentrations to Der p 1 in floor dust samples was only moderate and similar to the correlation between Der f 1 and Der p 1 (Pearson of log-transformed values r = 0.58 and 0.56, respectively), they were also well correlated to the sums of Der f 1 and Der p 1 (Pearson of log-transformed values r = 0.83). We conclude that the main allergen Der f 1 is a relatively constant proportion of total domestic mite antigens that ranged from 0.3 to 2.2% for 90% of the double positive samples. In line was also the mean factor of 144 between the Der f 1 and DM-antigen content of samples and the amount of 0.7% Der f 1 in the *D. farinae* standard of the DM-EIA.

Proportionality of DM antigen and Der f 1 values was true for both floor dust and airborne dust, with the limitation that only six airborne samples had detectable levels of Der f 1. Therefore, the DM-EIA and, especially, the DM-FEIA, with their much higher number and rate of measurable airborne samples (80 positives, 87%), should be valuable tools for quantification of airborne domestic mite antigens and thus, estimation of allergen exposure.

For floor dust samples, the DM-EIA had sufficient sensitivity; all samples were above the detection limit. Most workplaces had significantly lower DM concentrations per gram of dust than living areas, with the exception of textile recycling, bed feather cleaning and tailoring. Nevertheless, only floor dust from textile recycling plants had significantly higher DM concentrations in dust than living areas. As expected from the high correlation between the DM and Der f 1 values, the floor dust results are affirmed by the Der f 1 measurements. Again, only textile recycling had significantly higher values, whereas nearly all other workplaces had significantly lower allergen concentrations than living areas. The latter results are in line with studies from Italy in which Der f 1 and Der p 1 allergens were detected with low frequencies in schools and workplaces and with high frequency in homes [21]. However, inhalable airborne mite antigen concentrations displayed a different pattern. At most workplaces, a higher exposure occurred than in living areas. These differences were significant for textile recycling, bed feather filling, feed production, grain storage and cattle stables. One reason for higher inhalable airborne antigen concentrations may lie in greater air disturbances at workplaces producing significantly higher concentrations of airborne dust compared to living areas. Especially during feather filling of bed inlets using an air blower, dust and mite antigen exposure was significantly increased, although antigen concentrations in floor dust were lower than in living areas. Using the DM-FEIA, 87% of the airborne samples contained measurable domestic mite antigen concentrations, whereas only 6 samples (6.5%) contained Der f 1, and one sample Der p 1 (1.1%). Low concentrations of group I and group II mite allergens in airborne samples have been described previously and were explained by the relatively larger sizes of mite allergen-carrying particles when compared to the ones carrying the main cat allergen Fel d 1 [22]. The authors stated that particles with mite allergens only become airborne during disturbance and fall within 15 minutes. Quantification of single airborne mite allergens without vigorous air disturbances needed hitherto even with amplified or radioactive assay variants sampling times >24 hours [23]–[25]. The newly developed DM-assays with lower specificity enabled mite antigen quantification in personal dust samples using sampling times of 2–4 h during usual activities in household and at workplace.

## Conclusions

Overall, the newly developed DM-EIA and DM-FEIA are valuable tools to measure airborne domestic mite antigen exposure. The results of our first measurements of personal inhalable antigen exposure indicate that at some workplaces, such as textile recycling, higher inhalable domestic mite antigen concentrations occur than during house work. Further studies on domestic mite antigen exposure (e.g., in daycare centers) are planned.

## References

[1] Heinzerling LM, Burbach GJ, Edenharter G, Bachert C, Bindslev-Jensen C, et al (2009) GA(2)LEN skin test study I: GA(2)LEN harmonization of skin prick testing: novel sensitization patterns for inhalant allergens in Europe. Allergy 64: 1498–1506. 19772515 10.1111/j.1398-9995.2009.02093.x

[2] Arshad SH (2010) Does exposure to indoor allergens contribute to the development of asthma and allergy? Curr Allergy Asthma Rep 10: 49–55. 20425514 10.1007/s11882-009-0082-6

[3] Brussee JE, Smit HA, van Strien RT, Corver K, Kerkhof M, et al (2005) Allergen exposure in infancy and the development of sensitization, wheeze, and asthma at 4 years. J Allergy Clin Immunol 115: 946–952. 15867850 10.1016/j.jaci.2005.02.035

[4] Carlsten C, Ferguson A, Dimich-Ward H, Chan H, DyBuncio A, et al (2011) Association between endotoxin and mite allergen exposure with asthma and specific sensitization at age 7 in high-risk children. Pediatr Allergy Immunol 22: 320–326. 21255084 10.1111/j.1399-3038.2010.01123.x

[5] Gehring U, Heinrich J, Jacob B, Richter K, Fahlbusch B, et al (2001) Respiratory symptoms in relation to indoor exposure to mite and cat allergens and endotoxins. Indoor Factors and Genetics in Asthma (INGA) Study Group. Eur Respir J 18: 555–563. 11589355 10.1183/09031936.01.00096801

[6] Antens CJ, Oldenwening M, Wolse A, Gehring U, Smit HA, et al (2006) Repeated measurements of mite and pet allergen levels in house dust over a time period of 8 years. Clin Exp Allergy 36: 1525–1531. 17177675 10.1111/j.1365-2222.2006.02603.x

[7] Tovey ER, Almqvist C, Li Q, Crisafulli D, Marks GB (2008) Nonlinear relationship of mite allergen exposure to mite sensitization and asthma in a birth cohort. J Allergy Clin Immunol 122: 114–8, 118. 10.1016/j.jaci.2008.05.010 18602569

[8] Zock JP, Heinrich J, Jarvis D, Verlato G, Norbäck D, et al (2006) Distribution and determinants of house dust mite allergens in Europe: the European Community Respiratory Health Survey II. J Allergy Clin Immunol 118: 682–690. 16950288 10.1016/j.jaci.2006.04.060

[9] Paufler P, Gebel T, Dunkelberg H (2001) Quantification of house dust mite allergens in ambient air. Rev Environ Health 16: 65–80. 11354542 10.1515/reveh.2001.16.1.65

[10] Custovic A, Simpson B, Simpson A, Hallam C, Craven M, et al (1999) Relationship between mite, cat, and dog allergens in reservoir dust and ambient air. Allergy 54: 612–616. 10435476 10.1034/j.1398-9995.1999.00062.x

[11] Zahradnik E, Sander I, Flagge A, Fleischer C, Schierl R, et al (2009) Quantification of cow hair allergens with a two-sided enzyme immunoassay. Allergy 64: 171.

[12] Sander I, Flagge A, Merget R, Halder TM, Meyer HE, et al (2001) Identification of wheat flour allergens by means of two-dimensional immunoblotting. J Allergy Clin Immunol 107: 907–913. 11344361 10.1067/mai.2001.113761

[13] Thomas WR (2010) Geography of house dust mite allergens. Asian Pac J Allergy Immunol 28: 211–224. 21337903

[14] Spieksma FT (1991) Domestic mites: their role in respiratory allergy. Clin Exp Allergy 21: 655–660. 1777827 10.1111/j.1365-2222.1991.tb03192.x

[15] Heymann PW, Chapman MD, Aalberse RC, Fox JW, Platts-Mills TA (1989) Antigenic and structural analysis of group II allergens (Der f II and Der p II) from house dust mites (Dermatophagoides spp). J Allergy Clin Immunol 83: 1055–1067. 2732406 10.1016/0091-6749(89)90447-8

[16] Heymann PW, Chapman MD, Platts-Mills TA (1986) Antigen Der f I from the dust mite Dermatophagoides farinae: structural comparison with Der p I from Dermatophagoides pteronyssinus and epitope specificity of murine IgG and human IgE antibodies. J Immunol 137: 2841–2847. 2428875

[17] Barber D, Arias J, Boquete M, Cardona V, Carrillo T, et al (2012) Analysis of mite allergic patients in a diverse territory by improved diagnostic tools. Clin Exp Allergy 42: 1129–1138. 22702511 10.1111/j.1365-2222.2012.03993.x

[18] Ebner C, Feldner H, Ebner H, Kraft D (1994) Sensitization to storage mites in house dust mite (Dermatophagoides pteronyssinus) allergic patients. Comparison of a rural and an urban population. Clin Exp Allergy 24: 347–352. 8039020 10.1111/j.1365-2222.1994.tb00245.x

[19] van der Heide S, Niemeijer NR, Hovenga H, de Monchy JGR, Dubois AEJ, et al (1998) Prevalence of sensitization to the storage mites Acarus siro, Tyrophagus putrescentiae, and Lepidoglyphus destructor in allergic patients with different degrees of sensitization to the house-dust mite Dermatophagoides pteronyssinus. Allergy 53: 426–430. 9574887 10.1111/j.1398-9995.1998.tb03917.x

[20] Sun BQ, Lai XX, Gjesing B, Spangfort MD, Zhong NS (2010) Prevalence of sensitivity to cockroach allergens and IgE cross-reactivity between cockroach and house dust mite allergens in Chinese patients with allergic rhinitis and asthma. Chin Med J (Engl) 123: 3540–3544. 22166627

[21] Brunetto B, Barletta B, Brescianini S, Masciulli R, Perfetti L, et al (2009) Differences in the presence of allergens among several types of indoor environments. Ann Ist Super Sanita 45: 409–414. 20061661 10.1590/s0021-25712009000400009

[22] de Blay F, Heymann PW, Chapman MD, Platts-Mills TA (1991) Airborne dust mite allergens: comparison of group II allergens with group I mite allergen and cat-allergen Fel d I. J Allergy Clin Immunol. 88: 919–926. 10.1016/0091-6749(91)90249-n 1744363

[23] Sakaguchi M, Inouye S, Yasueda H, Irie T, Yoshizawa S, et al (1989) Measurement of allergens associated with dust mite allergy. II. Concentrations of airborne mite allergens (Der I and Der II) in the house. Int Arch Allergy Appl Immunol 90: 190–193. 2583857 10.1159/000235022

[24] Sakaguchi M, Inouye S, Sasaki R, Hashimoto M, Kobayashi C, et al (1996) Measurement of airborne mite allergen exposure in individual subjects. J Allergy Clin Immunol 97: 1040–1044. 8626979 10.1016/s0091-6749(96)70255-5

[25] Glasgow NJ, Ponsonby AL, Kemp A, Tovey E, van AP, et al (2011) Feather bedding and childhood asthma associated with house dust mite sensitisation: a randomised controlled trial. Arch Dis Child 96: 541–547. 21451166 10.1136/adc.2010.189696 PMC3093241

